# Autologous Peritoneum Graft Repair of a Superior Mesenteric Vein Defect During Pancreaticoduodenectomy

**DOI:** 10.7759/cureus.340

**Published:** 2015-10-02

**Authors:** Cuneyt Kayaalp, Fatih Sumer, Yilmaz Polat, Ramazan Kutlu

**Affiliations:** 1 Department of Surgery, Inonu University; 2 Department of Radiology, Inonu University

**Keywords:** pancreatic cancer, pancreaticoduodenectomy, superior mesenteric vein, peritoneum, vascular surgery

## Abstract

Pancreatic cancers frequently invade the portomesenteric veins. Venous resection during pancreaticoduodenectomy with curative intent is more common now than it was in the past. Most venous resections can be repaired primarily, but some require vascular grafts. Here, we describe the use of an autologous parietal peritoneum graft instead of vascular grafts for repairing a superior mesenteric vein (SMV) defect. Pylorus-preserving pancreaticoduodenectomy combined with en bloc resection of the SMV lateral wall was performed in a 70-year-old woman with cancer of the pancreatic head. The SMV defect was 2 cm long and its width was half the SMV circumference. The defect was covered with a 3 × 2 cm parietal autologous peritoneum graft obtained from the left subcostal area and using running 6/0 polypropylene suture. Tension-free patching was performed, and we retained slight bulging of the graft. Her postoperative course was uneventful. She was discharged on Day 11 after computed tomography confirmed the patency of the SMV, despite slight narrowing. She was well after 10 months of follow-up. Autologous parietal peritoneum grafts can be used for repairing partial venous defects during pancreaticoduodenectomy. They are effective and are easy, fast, and cheap to obtain.

## Introduction

The most effective treatment for malignant periampullary tumors is curative resection by pancreaticoduodenectomy. Although the invasion of the portomesenteric veins by periampullary cancers is common, en bloc venous resection in addition to pancreaticoduodenectomy does not compromise the oncological results. Most venous resections can be reconstructed primarily, but some require vascular grafts. The options for repairing portomesenteric venous defects are synthetic, autologous, or cryopreserved homologous vascular grafts. Here, we describe the use of an autologous parietal peritoneum graft for superior mesenteric vein (SMV) grafting after partial resection during pancreaticoduodenectomy.

## Technical report

Informed patient consent was obtained for this patient's treatment. No identifying patient information was used in this study.

**Figure 1:**
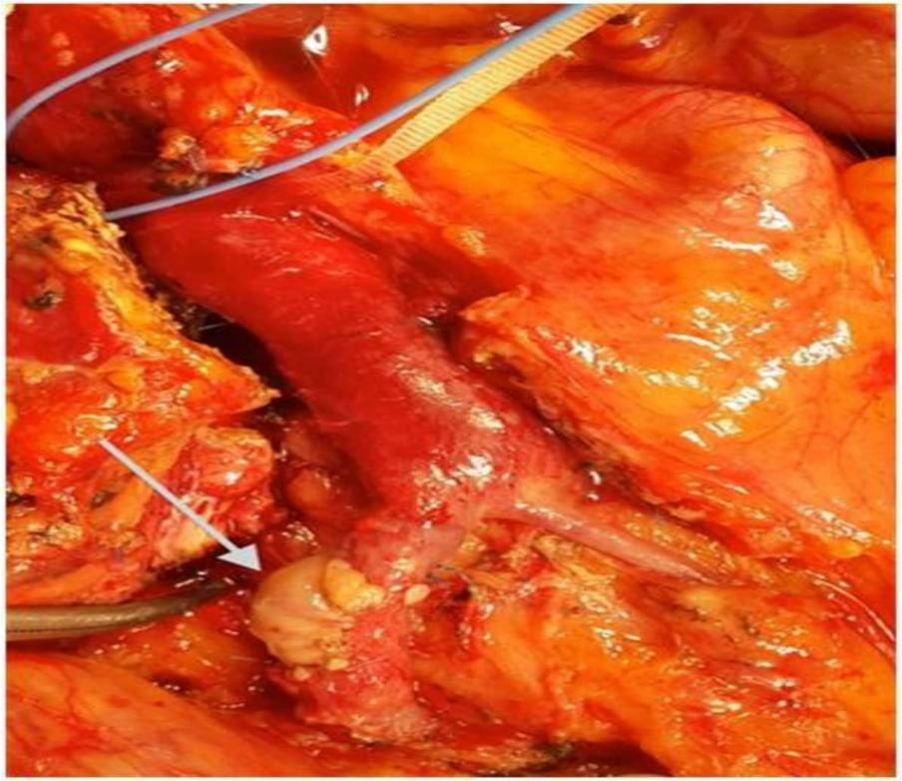
A 3 x 2 cm section of the parietal peritoneum from the patient was used to cover the superior mesenteric vein defect. We left a bulging of the graft on the vein.

A 70-year-old woman with Parkinson’s disease was referred to our center for cancer of the pancreatic head. She had no jaundice but had abdominal pain and weight loss for two months. Computed tomography (CT) revealed a mass (3 × 4 cm) at the inferior portion of the pancreatic head. CT also revealed that there was no clear path between the mass and the portomesenteric veins, dilated common bile duct (35 mm), and no distant metastasis. Her laboratory results, including tumor markers, were in the normal ranges. We did not perform a preoperative biopsy or administer neoadjuvant therapy, and surgical exploration was planned. Laparotomy confirmed the mass at the inferior part of the pancreatic head and that there was no distant metastasis, only local tumor invasion to the lateral border of the SMV. Pylorus-preserving pancreaticoduodenectomy and en bloc resection of the SMV lateral wall was performed. The SMV defect began 1 cm away from the portal–splenic vein confluence. The defect was 2 cm long and its width was half of the SMV circumference. A 3 × 2 cm section of the parietal peritoneum was removed from the left subcostal area of the patient and used to repair the defect by running 6/0 polypropylene suture. Tension-free patching was performed and we retained a slight bulging of the graft on the SMV (Figure 1).

**Figure 2:**
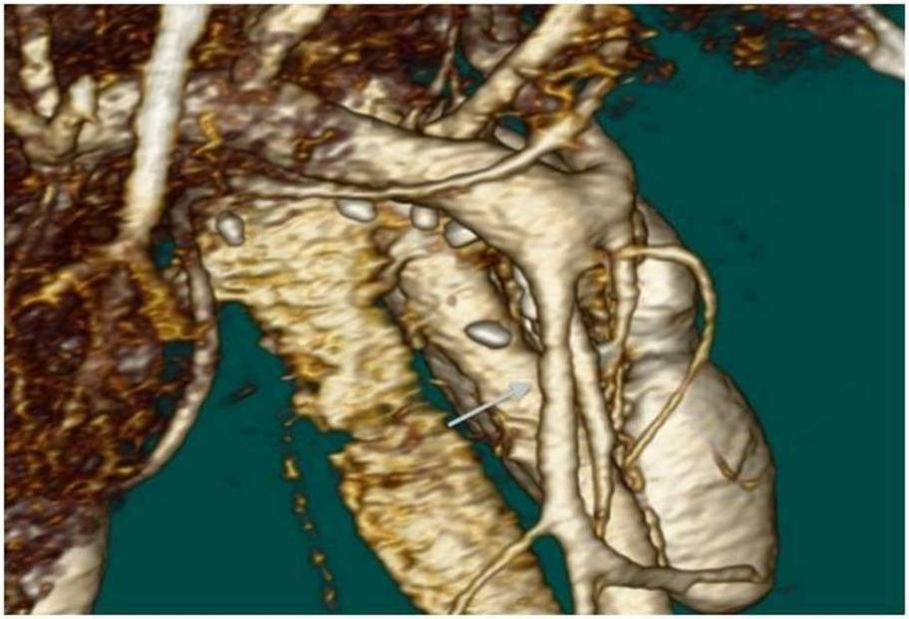
Contrast-enhanced 3D computed tomography of the patient on day 11. The superior mesenteric vein was patent.

The pancreatic stump was anastomosed by Wirsung jejunostomy over an internal stent, and other anastomoses (hepaticojejunostomy and duodenojejunostomy) were performed routinely. Operating time was five hours with a 200 ml blood loss. Her postoperative course was uneventful except for atelectasis, which was treated by chest physiotherapy. Her anticoagulation therapy was started on postoperative day 1 and continued throughout her hospital stay. She was discharged on postoperative day 11 after CT confirmed the patency of the SMV (Figure 2).

Histopathology detected adenosquamous carcinoma of the pancreas (4.8 cm in diameter), and 10 of the resected 31 lymph nodes were positive for metastasis. Combined chemoradiotherapy was started. She was well after 10 months of follow-up.

## Discussion

Peritoneum grafts appear to be safe, effective, easy to obtain, and cheap for repairing partial portomesenteric venous defects. Synthetic grafts for venous substitution have drawbacks, such as suturing difficulties due to their thin, non-pliable walls, and can be costly. Obtaining autologous grafts requires additional visceral dissection (renal or iliac veins), and additional incisions (neck or leg) may occasionally be necessary. Cryopreserved veins cannot always be prepared in time for surgery, their endothelium can be damaged over time, and thawing can be time-consuming. By contrast, a peritoneum graft of suitable size can be obtained more quickly than the above-mentioned options. Rapid acquisition is particularly important in conditions when emergency vascular reconstruction is necessary [1].

**Figure 3:**
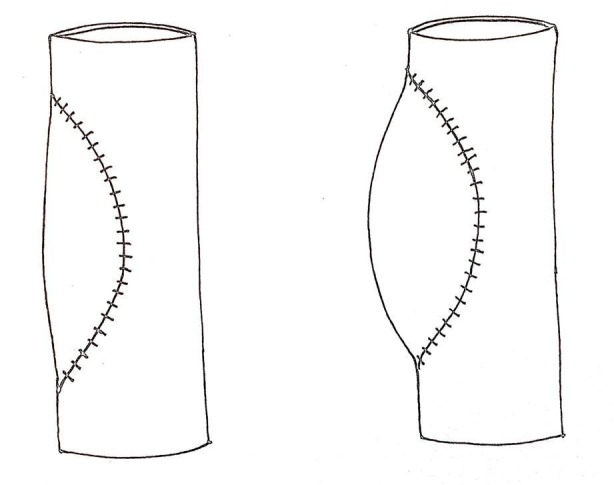
A bulging graft should be preferred to straight patching.

We have experience using the peritoneum for vascular grafts in living donor liver transplantation [1-2]. Our first case was a liver donor, whose left hepatic vein was repaired using a peritoneal graft under emergency conditions [1]. The second case was a living donor liver transplant recipient who required an 8 × 3 cm graft for repairing the extrahepatic venous conduit at the cut surface of the liver graft [2]. Both cases had successful outcomes; we subsequently decided to use peritoneal grafts for portomesenteric venous repair. Yoshioka first described the reconstruction of portomesenteric vein defects using peritoneum grafts in a porcine model in 2001 [3]. That experimental study demonstrated that the patency rates of the veins were good and that the risk of infection was low. Interestingly, the animals did not receive any postoperative antibiotics, anticoagulants, antithrombotics, or anti-platelet drugs [3]. Recently, Dokmak and co-workers reported the peritoneal grafting of several hepato-bilio-pancreatic venous defects in 30 patients [4]. In that study, all of the veins were larger than the SMV (portal vein, hepatic vein, or vena cava). There was none to mild stenosis in 90%, moderate stenosis in 7%, and thrombosis in 3% of those patients. Although bulging tends to be followed by venous thrombosis, the graft will retract in time and shrinking will occur. Therefore, we believe that vein grafting that retains a slight bulge in the peritoneum graft can prevent vascular stenosis (Figure 3). 

## Conclusions

Autologous parietal peritoneum grafts could be used for repairing partial venous defects during pancreaticoduodenectomy, and slight bulging of the graft appears necessary for preventing stenosis. 

## References

[1] Yilmaz S, Kayaalp C, Battaloglu B, Ersan V, Ozgor D, Piskin T (2012). Hepatic vein stenosis developed during living donor hepatectomy and corrected with peritoneal patch technique: a case report. Transplant Proc.

[2] Kayaalp C, Abbasov P, Sabuncuoglu MZ, Alam AH, Yilmaz S (2015). Peritoneal patch for an occluded venous conduit of a right lobe during a living-donor liver transplant. Exp Clin Transplant.

[3] Yoshioka M, Onda M, Tajiri T, Akimaru K, Mineta S, Hirakata A, Takubo K (2001). Reconstruction of the portal vein using a peritoneal patch-graft. Am J Surg.

[4] Dokmak S, Aussilhou B, Sauvanet A, Nagarajan G, Farges O, Belghiti J (2015). Parietal peritoneum as an autologous substitute for venous reconstruction in hepatopancreatobiliary surgery. Ann Surg.

